# In Situ synNotch-Programmed Astrocytes Sense and Attenuate Neuronal Apoptosis

**DOI:** 10.3390/ijms26094343

**Published:** 2025-05-02

**Authors:** Shi-Yu Liang, Ling-Jie Li, Ya-Ru Huang, Jie Zhu, Fang Cui, Xiao-Yu Du, Lun Zhang, Ying-Bo Jia, Sheng-Jie Hou, Xiao-Yun Niu, Jin-Ju Yang, Shuai Lu, Rui-Tian Liu

**Affiliations:** 1 State Key Laboratory of Biopharmaceutical Preparation and Delivery, Institute of Process Engineering, Chinese Academy of Sciences, Beijing 100190, China; liangshiyu20@ipe.ac.cn (S.-Y.L.); lilingjie@ipe.ac.cn (L.-J.L.);; 2 University of Chinese Academy of Sciences, Beijing 100049, China

**Keywords:** neuronal apoptosis, phosphatidylserine (PS), reprogrammed astrocyte, synNotch receptor, gaussia luciferase (GLuc), brain-derived neurotrophic factor (BDNF)

## Abstract

Neuronal apoptosis is an early and critical pathological hallmark of many chronic neurodegenerative diseases, often occurring silently long before the appearance of overt clinical symptoms. In this study, we engineered astrocytes utilizing a dual-biomarker recognition synNotch system (dual-synNotch). This system is designed to specifically identify neuronal apoptosis through the ‘AND Gate’ activation mechanism, which is triggered by the simultaneous sensing of the apoptotic signal phosphatidylserine (PS) and the neuronal signal ganglioside Gt1b. Upon detection of these neuronal apoptotic signals, the synNotch receptors are activated, inducing the expression of two key molecules: secreted Gaussia luciferase (GLuc), a highly detectable reporter that can cross the blood–brain barrier (BBB), and brain-derived neurotrophic factor (BDNF), a neuroprotective molecule that promotes neuronal survival by inhibiting apoptosis and enhancing memory and cognitive function. This engineered system effectively converts and amplifies early, imperceptible neuronal apoptotic signals into detectable outputs, enabling convenient in vitro monitoring and diagnosis. Therefore, it represents a promising strategy for the early detection and intervention of neurodegenerative diseases associated with neuronal apoptosis.

## 1. Introduction

Neurodegenerative diseases have emerged as the second leading cause of mortality, posing a significant threat to human health and life expectancy [1,2,3]. In the brain, neuronal death represents a core pathological hallmark of neurodegenerative diseases [4]. Neuronal cell death occurs extensively during development and pathology, where it is especially important because of the limited capacity of adult neurons to proliferate or be replaced. Apoptosis is a genetically controlled program of cell death by which a cell regulates its natural self-destruction. Neuronal apoptosis is thought to contribute to the development of a series of neurodegenerative diseases, including Alzheimer’s and Parkinson’s diseases [5]. Moreover, the progression and severity of neurodegenerative diseases are often closely linked to an increase in neuronal apoptosis [6]. Because of this leading role in neurodegenerative disorders, the major interest has traditionally been directed to elucidating the molecular mechanisms underlying neuronal apoptosis and devising strategies aimed at counteracting it.

Importantly, neuronal apoptosis often occurs long before the disease reaches a stage where it can be clinically diagnosed. Current diagnostic methods, such as positron emission tomography (PET) [7], single-photon emission computed tomography (SPECT) [8], magnetic resonance imaging (MRI) [9], plasma marker detection [10], fluorescent diagnostic probes [11], and clinical observation and cognitive assessments [12], primarily detect neurodegenerative diseases in their middle to late stages [13,14]. This diagnostic delay significantly narrows the therapeutic window and restricts the efficacy of available treatments. Additionally, many of these methods are time-intensive, costly, and sometimes invasive. Therefore, there is an urgent need to develop diagnostic methods that can detect neurodegenerative diseases at an earlier stage by detecting neuronal apoptosis using simpler, noninvasive, and more sensitive analytical tools.

One hallmark of apoptosis is the translocation of phosphatidylserine (PS) from the inner to the outer leaflet of the cell membrane, which serves as a key early indicator of apoptotic events [15,16,17]. To leverage this early apoptotic marker, apoptotic signals can be transduced into an alternative, quantifiable output, such as a highly sensitive luciferase signal, through the in situ modification of brain cells with a signal-sensing system [18,19,20]. Astrocytes, the most abundant glial cell type in the brain, play essential roles in promoting neuronal growth and survival and maintain intimate contact with neighboring neurons [21]. We are attempting to enable real-time and precise monitoring of neuronal apoptosis by in situ reprogramming of astrocytes with an apoptosis-sensing system. This approach allows for the real-time surveillance of neuronal apoptosis within the brain, providing timely alerts on apoptotic events during the initial stages of disease progression.

In the present study, we engineered the mouse Notch1 receptor (dual-synNotch) to function as a biosensor for neuronal apoptosis. This synthetic Notch1 (synNotch) receptor is designed to be effectively and precisely activated by cell surface antigens, with a robust ability to induce the expression of downstream genes, thereby initiating a cascade response targeting cell surface antigens [22,23,24]. This system is designed to specifically identify neuronal apoptosis through the ‘AND Gate’ activation mechanism, which is triggered by the simultaneous sensing of the apoptotic signal PS and the neuronal signal ganglioside Gt1b. Upon recognition of PS and Gt1b, the two receptors undergo enzymatic cleavage, releasing the cleaved intracellular transcription factors that subsequently mediate the transcription of corresponding target genes. In the present study, we chose PS as the apoptosis marker because of its well-established role as an indicator of cell apoptosis. Furthermore, Ganglioside Gt1b, a surface marker, was incorporated to distinguish neurons from other cell types, such as glial cells [25,26]. This dual-marker system effectively minimizes off-target activation and ensures that system activation is specifically linked to neuronal apoptosis. Upon ‘AND Gate’ activation, these astrocytes express Gaussia luciferase (Gluc), thereby reporting neuronal apoptosis. Furthermore, activated astrocytes can simultaneously express neurotrophic or neuroprotective factors such as brain-derived neurotrophic factor (BDNF), which supports neuronal survival and preserves neuronal function [27,28]. Thus, this engineered system facilitates both the precise detection and targeted alleviation of neuronal apoptosis, effectively combining diagnostic and therapeutic functions in a cascade-driven process.

## 2. Results

### 2.1. Design of an ‘AND Gate’ Dual-synNotch System for Sensing Neuronal Apoptosis

**Figure 1:**
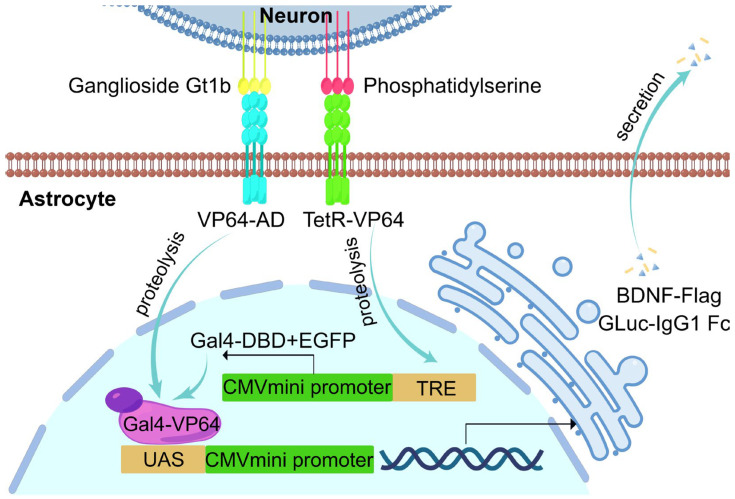
Schematic representation of the dual-synNotch system for sensing neuronal apoptosis. Astrocytes are genetically programmed to express two synNotch receptors on their surface, which specifically recognize the apoptosis marker PS and the neuronal marker ganglioside Gt1b. Upon activation, the synNotch receptor that binds to PS releases TetR-VP64 (tTA) into the nucleus, thereby initiating transcription of Gal4-DBD and EGFP. When activated by binding with Gt1b, the second synNotch receptor releases VP64-AD into the nucleus. VP64-AD then interacts with Gal4-DBD through complementary leucine zipper sequences to form a complete transcription factor, which subsequently initiates the transcription and expression of GLuc-IgG1 Fc and BDNF-Flag.

To specifically detect neuronal apoptosis, we designed a dual-biomarker recognition system, the dual-synNotch system, for expression on the membrane of astrocytes. This system enables simultaneous detection of the apoptotic marker PS and the neuronal marker ganglioside Gt1b (Figure 1). One of the synNotch receptors is activated upon recognizing PS through the Annexin A5 PS-binding domain [29,30], which is expressed at the N-terminal region of the synNotch receptor. This activation triggers the cleavage of the receptor, releasing its intracellular transcription factor, TetR-VP64 (tTA), into the cytoplasm. tTA then binds to the tetracycline response element (TRE), initiating transcription of downstream genes, including Gal4 DNA binding domain (Gal4-DBD) [31] and the reporter gene EGFP. The second synNotch receptor is engineered to recognize the neuron-specific biomarker ganglioside Gt1b via a tetanus toxin Gt1b-binding domain [32,33], which is also expressed at the N-terminal region of the receptor. Upon Gt1b recognition, this receptor releases a VP64 transcriptional activation domain (VP64-AD) motif from its intracellular segment. Subsequently, VP64-AD interacts with Gal4-DBD through a complementary leucine zipper structure [34,35], forming a complete transcription factor complex, Gal4-VP64. This complex then initiates expression of downstream proteins such as Gaussia luciferase (Gluc) and brain-derived neurotrophic factor (BDNF), driven by five copies of an upstream activating sequence (5× UAS). A mouse IgG1 Fc fragment conjugated with Gluc facilitates its crossing of the blood–brain barrier (BBB) into circulation and extending its half-life in the bloodstream by binding to FcRn receptors [36,37]. The Flag-tagged BDNF is anticipated to differ from wild-type BDNF and aims to attenuate neuronal apoptosis and promoting neuronal survival.

### 2.2. Kinetic Characterization of synNotch Receptor Activation

**Figure 2:**
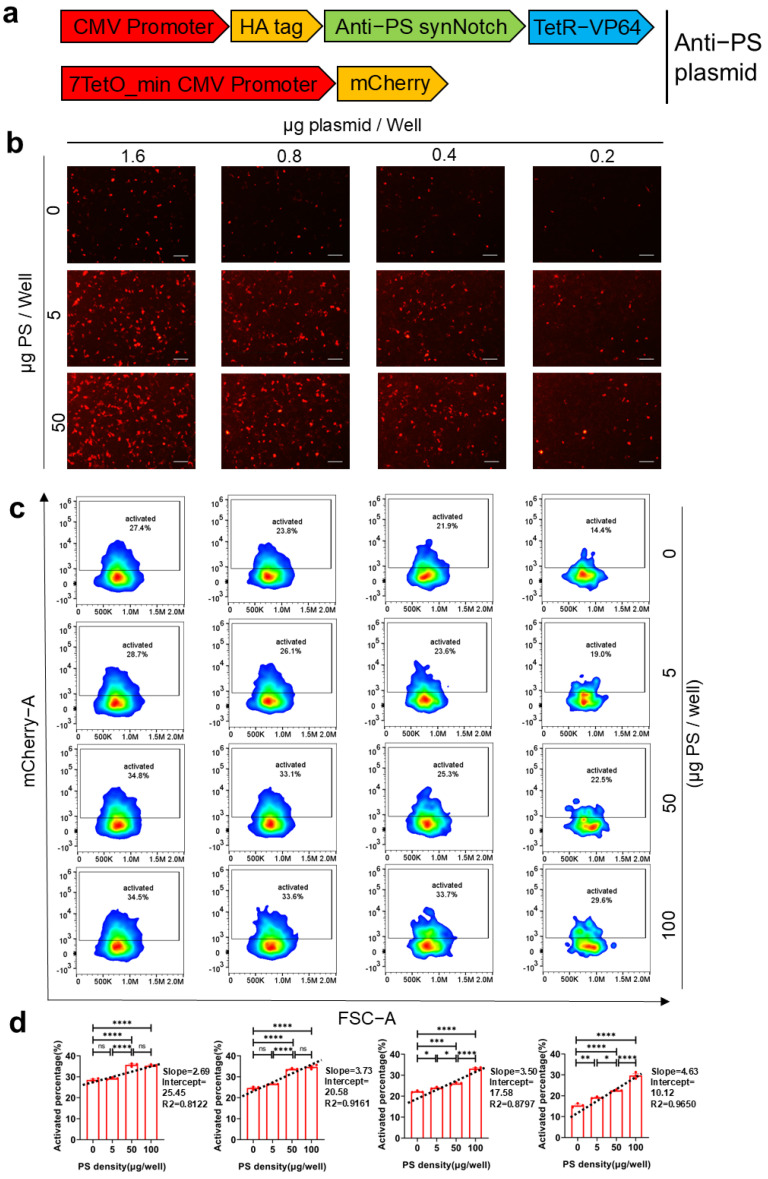
The effect of PS density and synNotch receptor density on activation. (**a**) Schematic illustration of the synNotch system for detecting PS. One plasmid encodes the gene sequences for the Annexin A5-synNotch receptor, which specifically recognizes the apoptotic signal PS. Additionally, it includes an inducible downstream protein reporter, mCherry, that is activated by the TetO promoter upon synNotch activation and subsequent release of TetR-VP64. (**b**) Representative images demonstrating synNotch activation in a COS7 cell model. COS7 cells were transiently transfected with varying amounts of plasmids as described in (**a**) and activated in the presence of 0, 5, and 50 µg PS per well. The activation of this system was visualized using mCherry fluorescence. Scale bar, 100 μm. (**c**) The ratio of COS7 cells expressing mCherry to the total number of COS7 cells exhibiting synNotch receptor expression was analyzed via flow cytometry. Colors represent cell density. (**d**) Ratios of mCherry-expressing COS7 cells in total synNotch-expressing COS7 cells cultured under different coating densities of PS and transfected with varying amounts of plasmids (*n* = 3 represents three independent experiments). The statistical points of each group were fitted by a dotted straight line. All data are shown as mean ± s.e.m. and were analyzed via one-way ANOVA with Tukey’s test (ns > 0.05; * *p* < 0.05; ** *p* < 0.01; *** *p* < 0.001; **** *p* < 0.0001).

To investigate the impact of the density of expressed synNotch receptors and their ligand PS on the activation of the synNotch system, various doses of PS, ranging from 0 to 50 μg/well, were incubated in the wells of cell culture plates. COS-7 cells were transfected with different amounts of plasmids per well to express synNotch receptors at varying densities on the cell surface and then incubated with the PS-containing wells. The results showed positive correlations between synNotch receptor activation and both the density of PS and the density of expressed synNotch receptors (Figure 2a–c). At a fixed receptor-expression density, a higher PS density promoted more robust system activation. Similarly, at a fixed PS density, an increased density of synNotch receptors led to stronger system activation; nevertheless, this increase also augmented the background activation of the system and reduced its sensitivity. At 0 μg/well PS density, the intensity of the mCherry signal served as an indicator of the system’s background noise. Moreover, the slope of the line between mCherry signal intensity and PS density reflected the sensitivity of the system. Lower synNotch receptor-expression densities, such as those achieved by transfecting 0.2 μg and 0.4 μg of plasmids per well, exhibited a steeper line slope, a lower background (as indicated by the intercept of the line), and a broader linear range of activation. In contrast, higher amounts of transfected plasmids, such as 0.8 μg and 1.6 μg per well, resulted in a higher activation background due to nonspecific receptor cleavage, along with a shallower line slope and a narrower linear range of activation (Figure 2d). Overall, transfection with 0.2 μg plasmids resulted in optimal system activation characteristics, with the highest sensitivity, lowest background noise, and the broadest linear activation range. Notably, the linear interval spanned from 0 to 100 μg PS per well, which was significant for the subsequent fitting and calibration of the antigen-activation curve.

### 2.3. Establishment of Detection Methods for Biomarker BDNF and Luciferase

**Figure 3:**
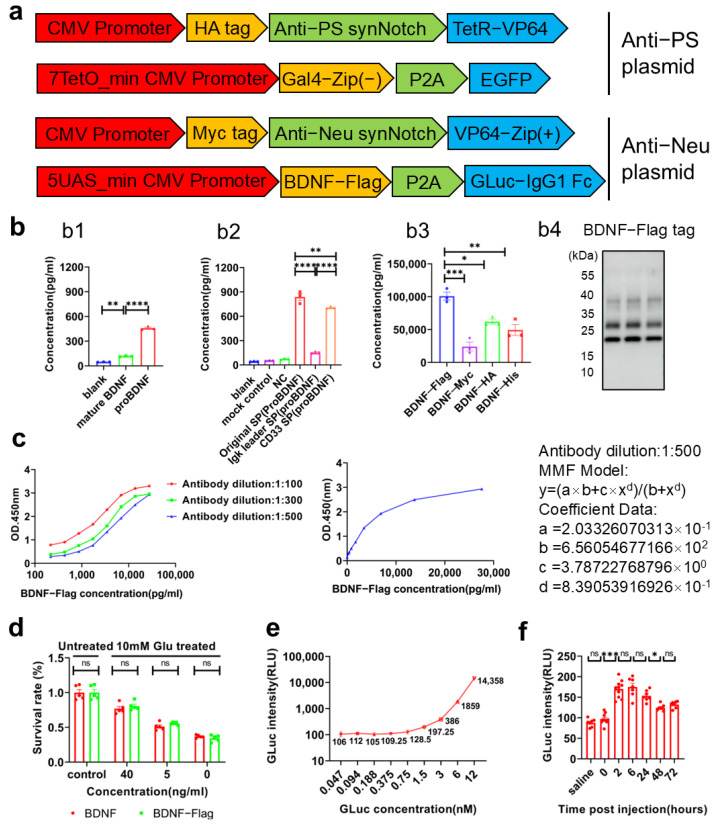
Detection of produced BDNF-Flag and Gluc-IgG1 Fc protein. (**a**) Schematic representation of the dual-synNotch system for detecting neuronal apoptosis. (**b**) Selection of BDNF types for expression. (**b1**) Comparison of the expression levels of BDNF precursor (proBDNF) and mature BDNF. (**b2**) Comparison of the expression levels of proBDNF with original signal peptide (sp), CD33 sp, and Ig κ leader sp. (**b3**) Comparison of the expression levels of proBDNF with different protein tags including Flag, Myc, HA, and His. (**b4**) The BDNF-Flag expressed by astrocytes in supernatant was detected by Western blotting using anti-Flag tag antibodies (*n* = 3 represents three independent experiments). (**c**) The standard ELISA curve of BDNF-Flag determined with various concentrations of BDNF-Flag under different dilution of detection antibody (left); curve fitting under 1:500 dilution of detection antibody through the MMF four-parameter fitting mode (**right**) (*n* = 3 represents three independent experiments). (**d**) The beneficial effect of BDNF-Flag on neurons. BDNF-Flag or wild-type BDNF protein were added to the 10 mM glutamic-acid-induced neuron apoptosis model, and the survival rate of neurons was detected by an MTT assay (*n* = 3 represents three independent experiments). (**e**) Standard curves for detection of GLuc-IgG1 Fc protein by measuring bioluminescence under different concentrations of GLuc-IgG1 Fc protein with the addition of coelenterazine (*n* = 3 represents three independent experiments). (**f**) Detection of GLuc-IgG1 Fc crossing the blood–brain barrier. The purified 1 μg GLuc-IgG1 Fc protein was injected into the unilateral lateral ventricle of the mouse brain, and the mouse serum was assayed at the indicated time points by measuring bioluminescence with the addition of coelenterazine (*n* = 7 represents seven independent experiments). All data are shown as mean ± s.e.m. and were analyzed via one-way ANOVA with Tukey’s test (ns > 0.05; * *p* < 0.05; ** *p* < 0.01;*** *p* < 0.001; **** *p* < 0.0001).

To specifically detect the BDNF generated upon activation, various tags were added to the C-terminal of BDNF. After experimental optimization based on protein expression, proBDNF fused with the Flag tag was selected (Figure 3b). To detect the recombinant BDNF-Flag, a sandwich ELISA method was developed. The anti-Flag monoclonal mouse antibody was used as a trapping antibody, and the anti-BDNF rabbit polyclonal antibody labeled with biotin was used as a detection antibody in the optimized ELISA system. This system was employed to measure the levels of BDNF-Flag in subsequent experiments with a minimum detection limit of 31.7 pg/mL (Figure 3c). Moreover, the BDNF protein fused with a Flag tag at its C-terminal retained its physiological function, as demonstrated by its similar beneficial effect on glutamic-acid-mediated neuronal apoptosis. These results indicate that fusing the Flag tag to BDNF does not affect the protein’s physiological function (Figure 3d).

To facilitate the easy and rapid detection of expressed neuronal apoptotic biomarkers, Gaussia luciferase (Gluc) was designed as a dedicated marker for highly sensitive detection [38]. For potential in vivo diagnostic applications, mouse IgG1 Fc was fused to the C-terminal of GLuc. This fusion enabled GLuc to cross the BBB into the bloodstream by binding to and then being released from the neonatal Fc receptor (FcRn) on the surface of brain vascular endothelial cells. The bioluminescence produced by the Gluc-IgG1 Fc fusion protein and its substrate, coelenterazine, exhibited an exponential growth relationship with the concentration of Gluc-IgG1 Fc, starting at 1.5 nM and above (Figure 3e). Upon injection into the mouse lateral ventricle, a portion of Gluc-IgG1 Fc successfully crossed the BBB into the blood circulatory system. We were able to detect GLuc-IgG1 Fc signal in the serum isolated from the venous blood of mice, confirming the ability of the fusion protein to traverse the BBB (Figure 3f).

### 2.4. Activation of synNotch Receptors by Coated PS and Gt1b

**Figure 4:**
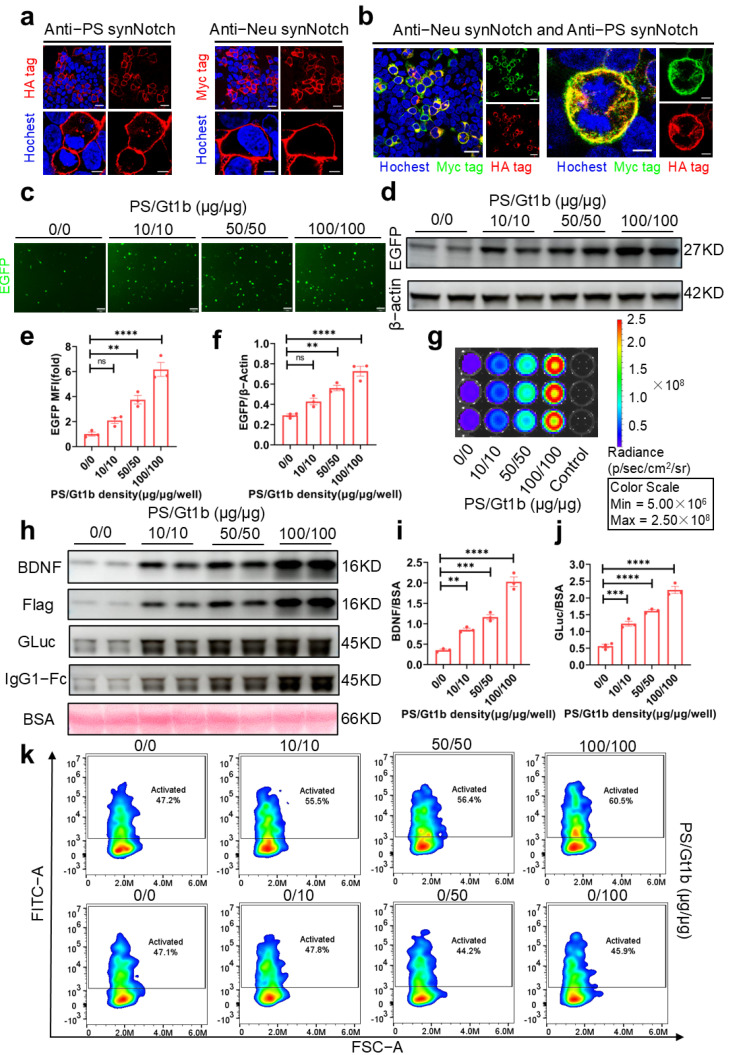
The expression of dual-synNotch receptors and biomarker production in HEK-293T cells. (**a**) Representative images illustrating the anti-PS synNotch receptor (**left**) and the anti-Neu synNotch receptor (**right**) expressed on the surface of HEK-293T cells. Scale bar, 25 μm, 5 μm in enlarged pictures. (**b**) Representative images showing the simultaneous expression of the anti-PS synNotch receptor (red) and the anti-Neu synNotch receptor (green) on the surface of HEK-293T cells. Scale bar, 25 μm, 5 μm in enlarged pictures. (**c**) Representative images showing the expression of reporter EGFP in HEK-293T cells expressing two receptors when the cells were simultaneously stimulated by different amounts of PS and ganglioside Gt1b for 24 h. Scale bar, 200 μm. (**d**) Western blotting analysis of EGFP in the cell lysate of HEK-293T stimulated with different amounts of PS and ganglioside Gt1b. (**e**,**f**) The mean fluorescence intensity (MFI) of EGFP (*n* = 3 represents three independent experiments) in (**c**) and the statistics of gray values of EGFP protein bands in (**d**) (*n* = 3 represents three independent experiments) were analyzed. (**g**) Bioluminescence image of the supernatants of the cell culture stimulated by different amounts of PS and ganglioside Gt1b following the addition of coelenterazine. (**h**) Western blotting analysis of BDNF-Flag and GLuc-IgG1 Fc in the supernatants of cell cultures stimulated with different amounts of PS and ganglioside Gt1b. (**i**) The statistics of gray values of BDNF-Flag protein bands were analyzed (*n* = 3 represents three independent experiments). (**j**) The statistics of gray values of GLuc-IgG1 Fc protein bands were analyzed (*n* = 3 represents three independent experiments). (**k**) Activated HEK-293T cells expressing EGFP in the presence of different amounts of PS and Gt1b analyzed by flow cytometry. Colors represent cell density. All data above are shown as mean ± s.e.m. and were analyzed via one-way ANOVA with Tukey’s test (ns > 0.05; ** *p* < 0.01; *** *p* < 0.001; **** *p* < 0.0001).

To validate the activation efficacy of the dual-synNotch system (Figure 3a), we first employed the HEK-293T cell model for our experiments. HEK-293T cells expressing the two synNotch receptors on their cell surfaces were cultured on plates precoated with PS and ganglioside Gt1b for 24 h (Figure 4a,b). Our findings showed that the coated PS and ganglioside Gt1b activated the two synNotch receptors. As the amounts of coated PS and ganglioside Gt1b increased, the mean fluorescence intensity (MFI) and expression level of the reporter protein EGFP enhanced (Figure 4c–f). Meanwhile, the expression level of secreted BDNF-Flag and GLuc-IgG1 Fc in the supernatants (Figure 4h–j) and the bioluminescence intensity of secreted GLuc-IgG1 Fc (Figure 4g) increased correspondingly. In contrast, an increase in ganglioside Gt1b alone did not activate the anti-PS synNotch receptors to produce the EGFP reporter (Figure 4k). These results indicated that the two synNotch receptors could be activated by the combination of the neuron marker Gt1b and the apoptosis marker PS, leading to the expression of the expected biomarkers in a signal intensity-dependent manner. Importantly, in the absence of the crucial apoptotic marker PS, the system could not be activated.

### 2.5. Activation of synNotch Receptors by Apoptotic Primary Neurons

**Figure 5:**
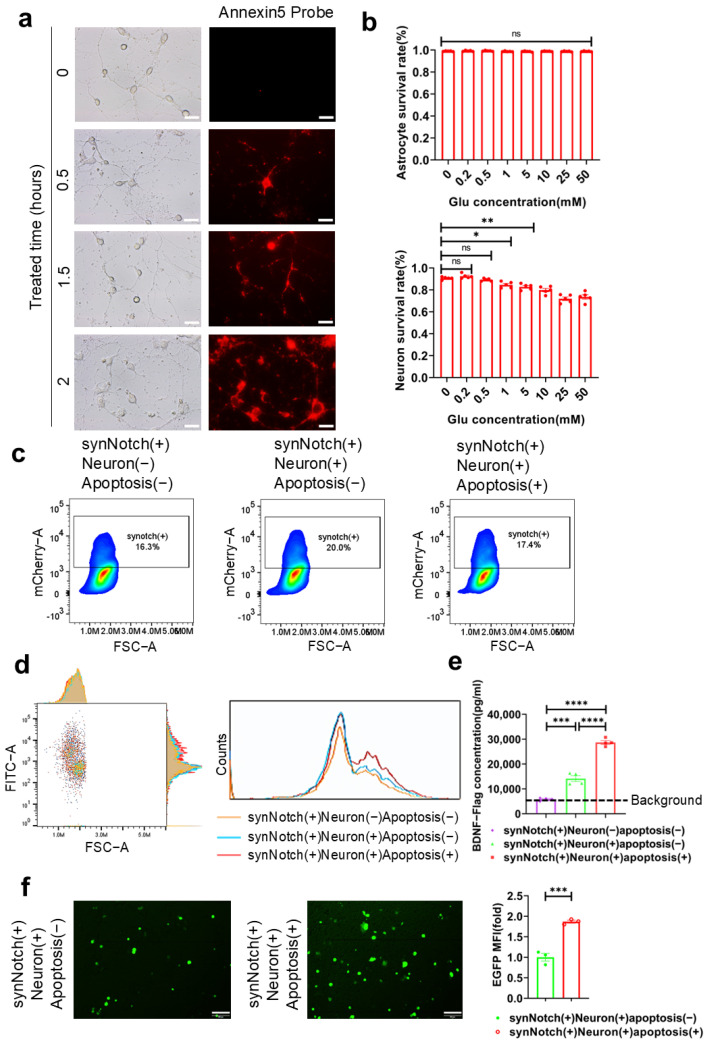
Activation specificity of synNotch system and construction of neuronal apoptosis model. (**a**) Representative images of primary neurons treated with 5 mM glutamic acid for different amounts of time and PS on neurons detected using an Annexin5-mCherry probe. Scale bar, 100 μm. (**b**) The apoptosis rates of neurons and astrocytes induced by different concentrations of glutamic acid were detected by TUNEL and Hochest costaining 6 h after glutamic acid addition and analyzed via one-way ANOVA with Tukey’s test (*n* = 5 represents five independent experiments). (**c**) Flow cytometry analysis of the total HEK-293T cells expressing synNotch receptors marked by mCherry. Colors represent cell density. (**d**) Flow cytometry analysis of the reporter EGFP in the dual-synNotch system expressed by HEK-293T cells, which were either activated by apoptotic neurons or not, for 24 h. (**e**) The concentration of BDNF-Flag in the supernatant of (**d**) was measured by ELISA and analyzed via one-way ANOVA with Tukey’s test (*n* = 3 represents three independent experiments). (**f**) Representative images of HEK-293T cells with reporter EGFP. The MFI of the reporter EGFP were counted and analyzed via a nonparametric unpaired *t* test with a two-tailed *p* value (*n* = 3 represents three independent experiments). Scale bar, 100 μm. All data above are shown as mean ± s.e.m. (ns > 0.05; * *p* < 0.05; ** *p* < 0.01; *** *p* < 0.001; **** *p* < 0.0001).

To confirm the binding between the synNotch receptors on the surface of HEK-293T cells and PS, as well as that of ganglioside Gt1b on the surface of apoptotic neurons, HEK-293T cells transfected with the two synNotch receptors were cocultured with mouse primary neurons. In accordance with previous reports [39,40], glutamic acid was added to the culture medium to induce neuronal excitotoxicity (Figure 5a,b). Within the concentration range of 0–50 mM, glutamic acid predominantly induced excitotoxicity of neurons without causing apoptosis of astrocytes. After only two hours of stimulation with 5 mM glutamic acid, a significant amount of PS on the surface of neurons could be visualized using the PS probe Annexin5-mCherry, indicating the significant occurrence of apoptosis. The HEK-293T cells were transfected with two plasmids as (Figure 3a) by liposome to express dual-synNotch receptors (Figure 5c). After incubation with glutamic-acid-treated neurons for 48 h, both the concentration of BDNF-Flag in the supernatant and the mean fluorescence intensity of EGFP increased significantly (Figure 5d–f). This suggests that neuronal apoptosis can effectively activate the synNotch receptors on HEK-293T cells and induce the expression of downstream markers. Although some background activation of HEK-293T cells cocultured with neurons in the absence of glutamic acid was observed, this might be attributed to normal apoptosis occurring within the neuronal culture. It is evident that neurons without glutamic acid induction still displayed a certain percentage of apoptosis (Figure 5b).

**Figure 6:**
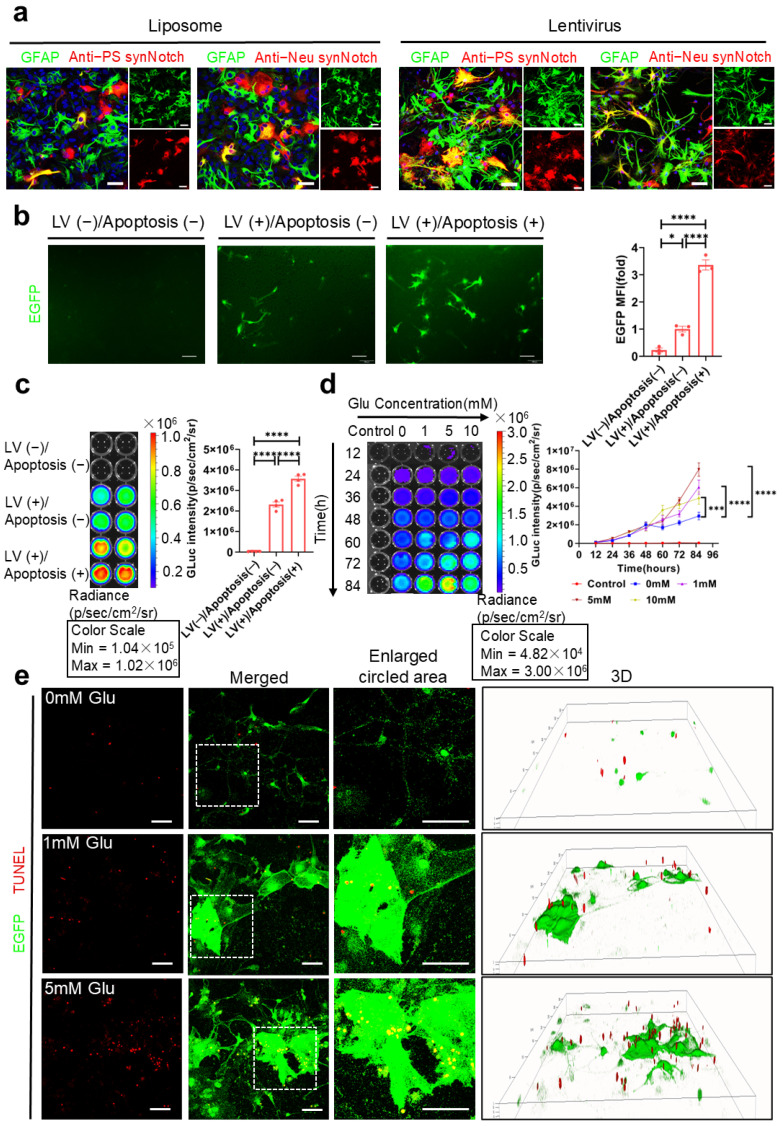
The activation of dual-synNotch receptors and biomarker production by primary astrocytes (**a**) Representative images of the two receptors expressed on primary astrocytes transfected by liposome and lentivirus. Scale bar, 50 μm. (**b**) Representative images of reporter EGFP in primary astrocytes. The primary astrocytes were transfected with double LVs (MOI = 1) and cocultured with neurons which were induced apoptosis by 5 mM L-glutamic acid for 48 h. Scale bar, 200 μm. The relative MFI of EGFP was analyzed via one-way ANOVA with Tukey’s test (*n* = 3 represents three independent experiments). (**c**) Bioluminescent imaging; The bioluminescent intensity of GLuc-IgG1 Fc in cell culture supernatants was measured in (**b**) (*n* = 4 represents four independent experiments) and analyzed via one-way ANOVA with Tukey’s test. (**d**) Bioluminescent imaging; The bioluminescent intensity of GLuc-IgG1 Fc in cell culture supernatants were measured at various time points after the induction of neuronal apoptosis in the presence of glutamic acid (*n* = 3 represents three independent experiments); the data were analyzed via two-way ANOVA with Tukey’s test. (**e**) Representative images of reporter EGFP in astrocytes and the TUNEL staining of neurons induced by 0, 1 and 5 mM glutamic acid for 48 h. Scale bar, 50 μm. All the above data are shown as mean ± s.e.m. (ns > 0.05; * *p* < 0.05; *** *p* < 0.001; **** *p* < 0.0001).

Under more physiologically relevant circumstances, we implemented the system in mouse primary astrocytes. In contrast to liposomes, the lentivirus (LV) vector exhibited a higher transfection efficiency in primary astrocytes cocultured with primary neurons, and we achieved the targeted expression of the synNotch receptor by employing the astrocyte-specific GfaABC1D promoter (Figure 6a). Astrocytes expressing the two synNotch receptors manifested a strong response to neuronal apoptosis 48 h after the addition of 5 mM glutamic acid. The levels of apoptosis-related biomarkers, such as EGFP and the secreted GLuc-IgG1 Fc in the supernatant, were significantly elevated in the apoptosis group as compared with the control group (Figure 6b,c). The activation strength of astrocytes increased in a manner that was reliant on both the concentration of glutamic acid and the progression of time (Figure 6d). Additionally, fluorescent immunocytochemistry analysis showed that the reporter protein EGFP was highly colocalized with TUNEL-positive cells, and the level of EGFP was positively correlated with the extent of neuronal apoptosis (Figure 6e). Moreover, we observed that the degree of neuronal apoptosis at the specified location, indicated by the area circled in Figure 6e, was positively correlated with the expression level of EGFP. This suggests that a higher density of neuronal apoptosis in this region corresponded to a more pronounced activation of astrocytes. These findings suggest that astrocytes reprogrammed with the two synNotch receptors via the LV vector specifically recognized apoptotic neurons and effectively transduced apoptotic signals into apoptosis-associated biomarker signals.

### 2.6. Transcriptome Characterization of Reprogrammed Astrocytes

Given that astrocytes provide crucial nutritional support for neurons [41] and play significant roles in synaptic formation [42] and other essential functions, these aspects cannot be overlooked. Thus, it is essential to determine whether the overexpression of the modified synNotch receptor in astrocytes has potential impacts on their own physiologically relevant functions. We measured the transcription levels of key genes associated with the normal physiological functions of astrocytes.

**Figure 7:**
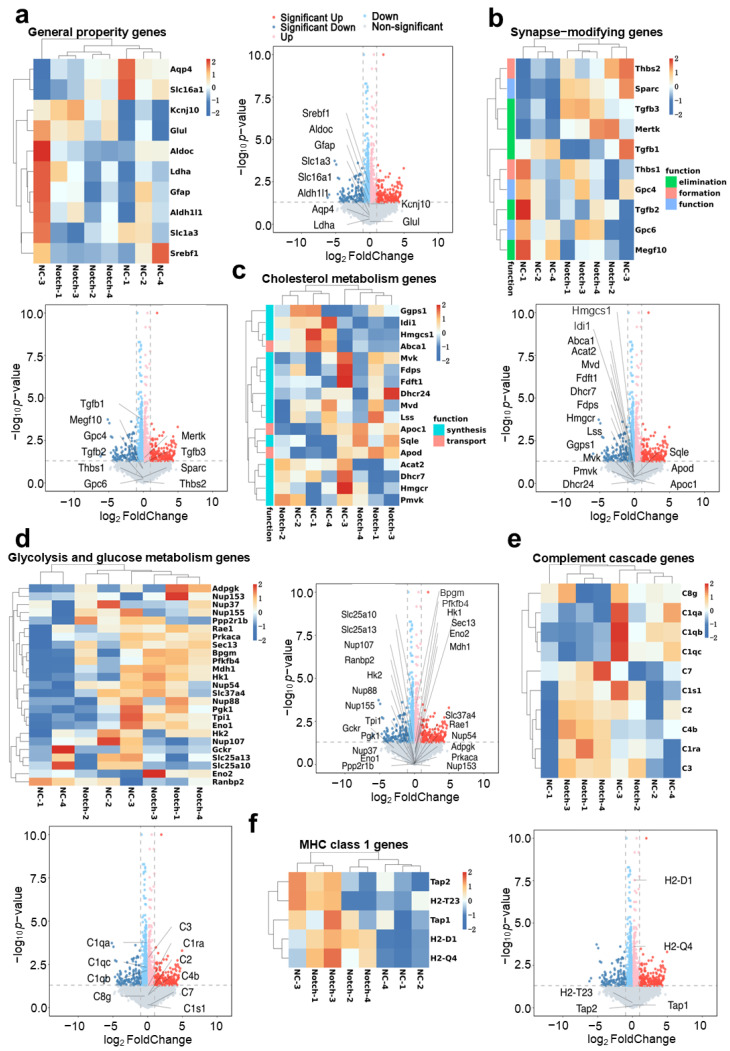
Transcriptome of neural support function, normal physiological metabolism, and immune response of reprogrammed primary astrocytes. (**a**) Cluster heatmap and volcano plot of general-property-related genes of astrocytes (*n* = 4 represents four independent experiments). (**b**) Cluster heatmap and volcano plot of synapse-modification-related genes of astrocytes (*n* = 4 represents four independent experiments). (**c**) Cluster heatmap and volcano plot of cholesterol-metabolism-related genes of astrocytes (*n* = 4 independent samples). (**d**) Cluster heatmap and volcano plot of glycolysis- and glucose-metabolism-related genes of astrocytes (*n* = 4 represents four independent experiments). (**e**) Cluster heatmap and volcano plot of complement-cascade-pathway-related genes of astrocytes (*n* = 4 represents four independent experiments). (**f**) Cluster heatmap and volcano plot of MHC-I-type-related genes of astrocytes (*n* = 4 represents four independent experiments). The grey vertical dotted line represents |log_2_ FoldChange|=1, and the grey horizontal line represents that *p*-value=0.05.

In the bulk RNA-seq analysis, no significant alterations (genes with a >2-fold change and *p* < 0.05) were detected in the majority of gene sets associated with the normal physiological functions of astrocytes. These included genes related to general properties, synapse-modifying genes, genes involved in glycolysis and glucose metabolism, and cholesterol metabolism genes. However, certain genes, such as Tgfb1 and Mertk, were modestly regulated. These findings indicated that the expression of the synNotch receptor did not impact the normal functions of astrocytes (Figure 7a–d). Additionally, the results revealed that most of the complement cascade genes associated with inflammation and the immune response, as well as MHC-I related genes, did not show significant changes (genes with a >2-fold change and *p* < 0.05). However, we did observe that certain genes, such as the complement-related C1 and C3 genes, along with the innate immunity related H2-D1 and H2-Q4 genes, were slightly regulated (Figure 7e,f). The analysis of these gene sets demonstrates that the expression of synNotch receptors and the corresponding intracellular activation pathway were orthogonal to the normal physiological genes’ pathways and did not affect the normal physiological function of astrocytes.

## 3. Discussion

Neuronal apoptosis often precedes the onset of clinical symptoms in neurodegenerative diseases. To enable early detection, we designed and optimized the synNotch system for reprogramming astrocytes. This system is activated when engineered astrocytes simultaneously detect apoptotic and neuronal signals, leading to the expression of Gaussia luciferase and BDNF, which not only reflect the extent of neuronal apoptosis but help mitigate its progression.

The selection of appropriate markers for detecting apoptosis is critical for the accurate identification of and response to apoptosis. In our study, PS was chosen as the apoptosis marker because of its well-established role as an indicator of cell apoptosis. Notably, PS externalization on the cell surface can also occur during cellular necrosis, indicating that synNotch receptor recognition of PS may extend to detecting other forms of cell death events such as necrosis [43,44] in necrosis-associated neurodegenerative diseases [45]. To enhance specificity, we incorporated Ganglioside Gt1b, a classic neuronal surface marker, to distinguish neurons from other cell types, such as glial cells. This dual-marker system effectively minimizes off-target activation and ensures that system activation is specifically linked to neuronal apoptosis.

The selection of receptors plays a pivotal role in determining the activation efficiency and sensitivity of the system. Compared with classical synthetic receptor platforms, such as chimeric antigen receptors (CAR) [46], modular extracellular signaling architecture (MESA) system [47], two-component systems (TCS) [48], and the Tango system [49,50], the synNotch receptor exhibits distinct advantages. Its activation pathway operates orthogonally to the cell’s intrinsic signaling pathways, thereby preserving normal cellular functions. Additionally, since the synNotch receptor is derived from the endogenous Notch1 receptor in mice, it can be efficiently expressed in murine-derived cells. The results revealed that the synNotch receptor system is also characterized by its relatively simple and efficient components and activation mode, making it an ideal candidate for achieving localized and precisely controlled cytokine secretion.

Beyond receptor selection, the modification and optimization of synNotch receptor can further enhance its activation efficiency and sensitivity. In recent years, the structural modification of synthetic receptors aiming at improving activation efficiency has emerged as a key focus in the field of synthetic biology. To increase the affinity of synNotch receptors for their target markers, we incorporated Annexin A5 and the C-terminal subunit of the tetanus toxin, which exhibit high affinity for PS and Gt1b, respectively. This selection was supported by the evidence that increased ligand–receptor affinity correlates with a heightened activation intensity of the synNotch receptor [23]. To further improve activation efficiency, we propose exploring single-chain variable fragments (scFv) or nanoantibodies with higher affinities for PS and Gt1b. Additionally, we enhanced receptor flexibility and binding capacity by linking the extracellular binding domain to the transmembrane domain using a flexible (GGGGS)_3_ linker. To avoid nonspecific receptor activation, we further incorporated an intracellular hydrophobic protein sequence (QHGQLWF) named RAM7 motif into the receptor structure [51]. Recent studies have also indicated that synthetic intramembrane proteolysis receptors (SNIPRs) generated via replacement of core fragments of synNotch may also enhance receptor activation efficiency [52].

In addition to the intrinsic factors related to receptor structure, we demonstrated that the receptor expression density significantly impacted both the activation intensity and system sensitivity (Figure 2b–d). While higher receptor density enhanced the activation intensity, it also increased nonspecific activation, thereby reducing the signal-to-noise ratio and the sensitivity of the system. Conversely, insufficient receptor density might result in the insufficient production of BDNF and luciferase, potentially falling below assay detection limits because of weak activation. Therefore, maintaining an optimal receptor density is crucial to balancing activation intensity and system sensitivity. In this study, we optimized transfection conditions to achieve enhanced sensitivity, reduced background noise, and a broader linear activation range. Importantly, the resulting BDNF and Gaussia luciferase levels met assay detection thresholds, enabling precise evaluation of neuronal apoptosis signals.

The mouse IgG1-Fc fragment, which is fused to the C-terminal of GLuc, facilitated GLuc’s crossing of the BBB into the bloodstream. By binding to the FcRn receptors on vascular endothelial cells, it also extended the half-life of GLuc, enabling more stable and convenient signal detection in bloodstream. We confirmed the expression of the GLuc-IgG1 Fc protein, its successful translocation across the BBB, and its prolonged half-life in mice (Figure 3e). Addtionally, BDNF, produced in response to neuronal apoptosis recognition, promotes neuronal survival and inhibits apoptosis, thus fulfilling dual functions of detection and therapy (Figure 3c). Given the dynamic equilibrium between BDNF in the central nervous system (CNS) and peripheral blood [53], measuring activated BDNF in peripheral blood could also serve as an indicator of neuronal apoptosis. However, detecting BDNF is inherently more complex and costly than measuring luciferase. To effectively distinguish activated BDNF from its wild-type form, we constructed a recombinant Flag tag fused BDNF protein (Figure 3a) and developed a high-sensitivity double-antibody sandwich ELISA detection system to enable precise quantification of activated BDNF (Figure 3b).

Through the transfection of the dual-synNotch system into HEK-293T cells and primary astrocytes, we demonstrated that this system could effectively recognize PS and ganglioside Gt1b, whether coated on cell culture plates or presented on the surface of apoptotic neurons. This recognition triggered dose-dependent activation, as depicted in Figure 4, Figure 5 and Figure 6. Increasing the concentration and duration of the apoptosis inducer, glutamic acid, significantly enhanced the extent of neuronal apoptosis (Figure 5a). Concurrently, both the GLuc-IgG1 Fc signal intensity and the BDNF concentration in the supernatant increased substantially, indicating that the system responded proportionally to the intensity of the apoptotic signal (Figure 5d–f and Figure 6b–d). Moreover, the activated astrocytes expressing the reporter protein EGFP showed a high degree of colocalization with TUNEL-positive apoptotic neurons, rather than with viable neurons, confirming the system’s high specificity (Figure 6e).

Regarding the safety of reprogramming astrocytes, we analyzed the transcriptomics of primary astrocytes in mice overexpressing synNotch (Figure 7). It is essential to ensure that the overexpression of heterologous synthetic receptors does not interfere with the intracellular pathways of reprogrammed cells. Compared with wild-type astrocytes, we observed no significant changes (genes with a >2-fold change and *p* < 0.05) in the expression of genes associated with key astrocyte functions, including neurotrophic support genes (Figure 7a,b), substance metabolism genes (Figure 7c,d), and immune response genes (Figure 7e,f). These findings support the safety and applicability of this in situ modification method. However, further in vivo validation and refinement in various disease models are needed to ensure its effectiveness and safety.

While the reprogrammed astrocytes successfully achieved neuronal apoptosis signal-dependent activation, we also observed some background activation, likely due to inherent nonspecific cleavage of the synNotch system. This phenomenon is an unavoidable characteristic of the synNotch receptors currently in use [51]. However, future studies should focus on modifying the synNotch receptor itself to reduce the background activation or enhance signal response intensity, thereby improving the signal-to-noise ratio for a more accurate indication of neuronal apoptosis. Additionally, minimizing the immunogenicity of both the synNotch receptor and its detection components will be crucial for clinical applications. Because of the considerable length of the genes encoding the entire dual-synNotch system, we opted for simultaneous delivery of two LVs, which slightly reduced the cotransfection efficiency of the system. It will be essential to further modify the entire system to reduce the size of gene fragments that need to be delivered. Ideally, these components should be constructed within a plasmid or viral vector concurrently, as this would significantly enhance the cotransfection efficiency of the system.

In summary, the study concluded that dual-synNotch system effectively translated the complex phenomenon of neuronal apoptosis into a specific and measurable signal, namely GLuc-IgG1 Fc, which was released into the bloodstream, enabling both the conversion and amplification of the neuronal apoptotic signal. Additionally, the BDNF produced by activation by neuronal apoptosis not only inhibited neuronal apoptosis but promoted neuronal survival. This innovative approach holds great potential for developing early-stage, convenient, and noninvasive diagnostic methods for diseases, especially for neurodegenerative disorders associated with neuronal apoptosis such as Alzheimer’s and Parkinson’s diseases.

## 4. Methods

### 4.1. Cell Lines

COS7 cells (ATCC, CRL-1651), HEK-293T cells (ATCC, CRL-3216), and HT22 cells (ATCC, LHY1277) were cultured in 10% fetal bovine serum (HUANKE) in DMEM (Invitrogen, 11965092) with 1% penicillin and streptomycin (Beyotime, C0222). All cell cultures were maintained in an incubator at 37% humidity and in a 5% CO_2_ atmosphere.

### 4.2. Plasmid Construction

Anti-PS synNotch receptors were built by fusing Annexin A5 (NM_001154, Met1-Asp320) to the mouse Notch1 extended regulatory region (NM_008714, Ile1427-Phe1759) with a (GGGGS)_3_ linker and then to TetR-VP64 (Addgene plasmid, Watertown, NY, USA, #79126). All anti-PS synNotch receptors contained a N-terminal CD8α signal peptide (N’-MALPVTALLLPLALLLHAARP-C’) with the aim of cell membrane localization and an HA tag epitope (N’-YPYDVPDYA-C’) for detecting the expression of the receptor by the anti-HA antibody (Abclonal, Wuhan, China, AE008). Anti-Neu synNotch receptors were built by fusing truncated tetanus toxin (X06214, Ile1111-Asp1315) to the mouse Notch1 extended regulatory region (NM_008714, Ile1427-Phe1759) with a (GGGGS)_3_ linker and then to VP64-Zip(+) (Addgene plasmid, #15305). All the anti-Neu synNotch receptors contained an N-terminal CD8α signal peptide (N’-MALPVTALLLPLALLLHAARP-C’) for cell membrane localization and an Myc tag epitope (N’-EQKLISEEDL-C’) for detecting the expression of the receptor with the anti-Myc antibody 9E10 (Santa Cruz, Dallas, TX, USA, sc-40, 1:200). The promoters on the 5′-terminal of synNotch receptor genes were a CMV promoter with a CMV enhancer or a GfaABC1D promoter followed by a Promega Chimeric Intron (Addgene plasmid, #100889). The response element downstream of the synNotch receptor contained an inducible promoter covering five copies of the Gal4 DBD targeting the UAS (5′-GGAGCACTGTCCTCCGAACG-3′) or seven copies of the TetR targeting the TRE sequence (5′-TCCCTATCAGTGATAGAGA-3′), followed by a minimal CMV promoter (Addgene plasmid, #79126). The reporter genes coding mCherry, EGFP, Gluc-IgG1 Fc (Addgene plasmid, #189629), and BDNF-Flag, which is a Flag tag (N’-DYKDHDGDYKDHDIDYKDDDDK-C’) directly linked to the C-terminal of mouse BDNF (NM_001048139, Met1-Arg249), were downstream of the inducible promoter. The BDNF-Flag contained a N-terminal original signal peptide (N’-MTILFLTMVISYFGCMKA-C’) that was optimized from the Igκ leader signal peptide (N’-METDTLLLWVLLLWVPGSTGD-C’) and the CD33 signal peptide (N’-MPLLLLLPLLWAGALA-C’). The gene fragment was obtained by PCR from Addgene plasmids (TaKaRa, R045Q). The whole genes were inserted into a pcDNA3.1 vector for liposome transfection or a pHR’SIN:CSW vector for lentiviral transduction using a Gibson Seamless Assembly cloning kit (Abclonal, RK21020).

In protein expression and purification experiments, plasmids encoding BDNF-Flag and Gluc-IgG1 Fc proteins were constructed by cloning the genes of mouse BDNF (NM_001048139, Met1-Arg249) with a Flag tag (N’-DYKDHDGDYKDHDIDYKDDDDK-C’) directly linked to the C-terminal and Gluc-IgG1 Fc (Addgene plasmid, #189629) into the pcDNA3.1 vector.

### 4.3. Lentivirus Production and Purification

The lentivirus packaging experiment was carried out with HEK-293T cells. The operations were as follows: First, HEK-293T cells were inoculated into a Petri dish coated with PDL. When the cell density reached about 70–90%, the cells were changed into a medium without antibiotics and continued to be cultured for 2 h. According to the proportion of PsPax2–PmD2.G–pHR’SIN:CSW = 2:1:3, three plasmids were mixed, and the mixture of plasmids and transfection reagent was added into the HEK-293T cells with Zlip2000 transfection reagent (Zomanbio, ZC305). After 6 h, the cell supernatant was removed, and a new medium was added for further culture. After 48 h of continuous culture, the supernatant of the cell culture containing the virus was collected for subsequent virus purification.

The cell culture supernatant containing the virus was centrifuged at 2000 rpm for 10 min, and the supernatant was collected and filtered with a 0.45 μm filter membrane. The virus supernatant after centrifugation was added to the centrifuge tube, and then 5 mL of 20% sucrose (PBS configuration) was slowly added to the bottom of the centrifuge tube. The centrifuge tubes were centrifuged at 25,000 rpm (SW28 rotor) at 4 °C for 3 h. After centrifugation, the supernatant was abandoned, and the virus precipitation was suspended with PBS and dissolved on ice for 2 h. The virus resuspension was collected and centrifuged at 4 °C at 16,000× *g* for 1 min. The supernatant was obtained as a lentiviral solution. Finally, the lentivirus solution was separated and stored in the refrigerator at −80 °C for later use.

### 4.4. Culture of Primary Neurons and Astrocytes

The brains of C57BL/6J fetal mice at E14 to E15 days of gestation were removed, the blood meninges were removed in HBSS, and the isolated hippocampus and cortex were cut up with tweezers. The tissue fragments were transferred to a 15 mL centrifuge tube and centrifuged at 800 rpm for 3 min. Then, 10 mL 0.25% pancreatic enzyme digestion solution (Beyotime, Shanghai, China, C0201) including DNase I (Solarbio, Beijing, China, D8071) was added to the precipitation, digested at 37 °C for 10 min, and gently reversed several times every 5 min. After digestion, the pancreatic enzyme digestion reaction was terminated with 20 mL DMEM medium (Gibco, Grand Island, NY, USA, 11965092) containing 10% FBS (Gibco, 10091155), the cells were gently blown into cell suspension with a pipette, and the undigested tissue mass was removed through a 70 μm cell screen. The remaining cells were collected by centrifugation at 1500 rpm for 10 min and then resuspended with DMEM medium and spread into 12-well plates. Basal culture medium with 2% B27 (Gibco, 17504044), 1% GlutaMAX (Gibco, 35050061), 0.5% penicillin, and streptomycin (Beyotime, C0222) was replaced with Neurobasal (Gibco, 21103049) medium for 2–4 h and continued for 7–9 days until neurons were mature for the follow-up study.

Primary astrocytes were extracted from the C57BL/6J neonatal mouse brain. The procedure was the same as that of primary neuron extraction. DMEM-F12 medium (Gibco, 11320033), containing 10% FBS and 1% penicillin and streptomycin, was cultured for 10–12 days, and the medium was changed every 2–3 days.

### 4.5. Plate Coated by PS and Gt1b

The indicated PS (YuanyeBio, Shanghai, China, S27340) and Gt1b (Cayman, Ann Arbor, MI, USA, 15588) dissolved by ultrasound in methanol (5 mg/mL) were added to 12-well polystyrene plates in various amounts and air-dried in a sterile ultraclean workbench for 2 h. The dried plates were used directly or stored at 4 °C.

### 4.6. Plasmid Transfection and Lentiviral Transduction In Vitro

Plasmids coding anti-PS synNotch and anti-Neu synNotch were at a 1:1 ratio in a total of 0.4 μg of plasmids per 2 × 10^5^ cells in a well of a 12-well plate with 2 μL of Zlip 2000 transfection reagent. After 6 h of incubation, the supernatant was removed and replaced with fresh medium for another 48 h. Then, the cells were used in later experiments. Lentiviral vectors coding anti-PS synNotch and anti-Neu synNotch were both added at an MOI = 1 per 2 × 10^5^ cells in a well of a 12-well plate. After 24 h of incubation, the supernatant was removed and replaced with fresh medium for another 48 h. Then, the cells were used in later experiments.

### 4.7. Cell Immunofluorescence Imaging and Analysis

The cell glass coverslips were washed three times with PBS for 5 min each time, fixed with 4% paraformaldehyde (Solarbio, P1110) at room temperature for 20 min, and then closed with a blocking solution containing 10% donkey serum (Solarbio, SL050) and 0.3% Triton-X100 (Sigma, St. Louis, MO, USA, T8787) for 1 h. Later, the corresponding primary antibody was added to the cell glass coverslips, and the coverslips were incubated at 4 °C overnight. Then, they were washed three times with PBS, the fluorescently labeled secondary antibody was added, and the coverslips were incubated at room temperature for 1 h. Hoechst 33258 was added and stained for 10 min at room temperature. After the film was sealed, the data were collected using confocal microscopy (Leica, Heerbrugg, Switzerland) and analyzed using the ImageJ (v1.8.0) software. The antibodies and dilutions used were as follows: mouse anti-HA antibody (Abclonal, AE008, 1:200), mouse anti-Myc antibody (Santa Cruz, sc-40, 1:100), rabbit anti-GFAP antibody (Affinity, Shanghai, China, DF6040, 1:200), and donkey antimouse, -rabbit, -chicken, and -goat Alexa fluor488, 546, 594, and 647 secondary antibodies (Life Technologies, Carlsbad, CA, USA, 1:500).

### 4.8. Flow Cytometry and Sorting

The cells were digested and suspended with 0.25% pancreatic enzyme, fixed with 4% paraformaldehyde for 2 min, and incubated at room temperature for 1 h with primary antibody. After the cells were washed three times with a flow buffer, which was PBS containing 0.1% Tween-20 (Beyotime, ST825) and 2% BSA (Beyotime, ST023), secondary antibody was added to the cells, and they were incubated at room temperature for 1 h. Then, after being washed with flow buffer for three times, the cells were resuspended with flow buffer and passed through a 100 μm cell screen (Beyotime, FSTR100). Data were then collected using CytoFLEX LX (Beckman, Pasadena, CA, USA) flow cytometry and analyzed with FlowJo (v10.8.1). SynNotch-positive astrocytes were sorted by a FACSAria II cell sorter for later RNA sequencing. The antibodies and dilutions used were as follows: mouse anti-HA antibody (Abclonal, AE008, 1:200), mouse anti-Myc antibody (Santa Cruz, sc-40, 1:100), and donkey antimouse Alexa fluor488 secondary antibodies (Life Technologies, 1:500).

### 4.9. BDNF-Flag Measurement by ELISA

Mouse anti-Flag antibody (Sigma, F1804) was coated on a 96-well plate with antibody coating buffer (2.935 g of NaHCO_3_ and 1.599 g of NaCO_3_ in 1 L of H_2_O at pH = 9.6), at a concentration of 0.5 μg/100 μL, and placed at 37 °C for 2 h. Then, the plate was washed with 0.05% PBST 3 times, sealing solution containing 0.05% PBST and 1% BSA was added in 300 μL per well, and the plate was sealed at 37 °C for 2 h. Later, the plate was washed with 0.05% PBST 3 times, the samples to be detected were added in 100 μL per well, and the plate was incubated at 37 °C for 1.5 h and washed with 0.05% PBST 3 times. Biotin-labeled rabbit antimouse BDNF (Signalway antibody, Wuhan, China, EK5128) diluted with sealing solution at a dilution of 1:500 was added at 100 μL per well, incubated at 37 °C for 1 h, and washed with 0.05% PBST for 3 times. Streptavidin-labeled HRP (Thermofisher, Waltham, MA, USA, 21140) diluted with sealing solution at a dilution of 1:5000 was added at 100 μL per well, incubated at 37 °C for 0.5 h, and washed with 0.05% PBST 5 times. TMB substrate solution (Solarbio, PR1210) was added at 100 μL per well and developed at 37 °C for 10 min. Finally, 1 M sulfuric acid termination solution was added at 100 μL per well to stop the chromogenic reaction. The absorption at 450 nm was measured by using a SpectraMax® M5 plate reader (Molecular Devices, Sunnyvale, CA, USA)within 5 min.

### 4.10. Protein Expression and Purification

BDNF-Flag protein was expressed by cultured primary astrocytes. Plasmids encoding three proteins were transfected into astrocyte cells, which were inoculated on 10 mm diameter cell culture dishes via Zlip2000 liposome. After 72 h of incubation, the cell culture supernatant was collected and centrifuged at 2000 rpm to remove cell debris. Next, 100 μL of magnetic beads coated with mouse anti-Flag tag antibody (MCE, Shanghai, China, HY-K0207) were incubated with 50 mL of cell culture supernatant at 4 °C on a shaker overnight. The next day, after the magnetic beads were separated from the cell supernatant using a magnetic rack, the magnetic beads were cleaned three times with 0.5% PBST until the OD_280_ in the supernatant after washing was less than 0.05. Then, 50 μL of elution buffer, which contained 0.15 M Glycine at pH = 2.5–3.1, was added to the magnetic beads, mixed evenly, and incubated together at room temperature for 10 min. The magnetic beads were separated, and the supernatant was collected into a new EP tube. The neutralization buffer, which contained 1 M Tris-HCl at pH = 8.0, was added at a ratio of 25 μL of neutralization buffer per 50 μL of eluent, and the pH of the elution product was soon adjusted to neutral. The eluted samples were stored at −80 °C or used for later functional analysis. The expression and purification of Gluc-IgG1 Fc were the same as the above process except that the magnetic beads were coated with protein-G (Beyotime, P2106) for the capture of the IgG1 Fc fragment.

### 4.11. Cytotoxicity Assay (MTT)

HT22 cells were cultured in DMEM containing 10% FBS and 1% penicillin/streptomycin. The cells in the Petri dish were digested by pancreatic enzymes, centrifuged, and then inoculated into 96-well plates with about 5000 cells per well per 100 μL of medium. After 12 h, the cells were treated with a 10 mM concentration of glutamate solution to induce neuronal apoptosis. After glutamate-induced neuronal apoptosis for 6 h, BDNF-Flag protein solution with a certain concentration gradient was added. After adding BDNF-Flag protein for 72 h, 25 μL 5 g/mL MTT (Solarbio, M8180) was added to each well, and the cells continued to culture at 37 °C for 3 h until the medium was absorbed. Later, 150 μL DMSO (Solarbio, D8371) was added to each cell well, and the reaction was performed at room temperature for 10 min away from light. The absorbance at 570 nm and 630 nm was measured using a microplate reader (MD-M5). There were six duplicate pore cells in each sample and control, and each experiment was repeated three times. After background correction, the relative cell survival rate was calculated by dividing the absorbance of the sample well by that of the control.

### 4.12. Brain Stereotaxic Injection

The mice were deeply anesthetized with a mixture of ketamine (100 mg/kg) and toluene thiazide (10 mg/kg) and fixed on a stereotaxic injection table. Bregma was used as the starting point using a stereotaxator with AP = −0.3 mm, ML = ±1.0 mm, DV = −1.8 mm for brain localization. After craniotomy with a cranial drill at a fixed point, injection was carried out with a microsyringe (Hamilton, Bonaduz, Switzerland, 10 μL), and a total of 1 μg GLuc-IgG1 Fc protein was injected into a single point at speed of 0.1 μL/min. After the injection, the needle was kept clean for 5 min so that the sample in the brain could be completely absorbed. The surgical site was cleaned with sterile saline, and the incisions were closed. The mice were monitored and provided with postoperative care.

### 4.13. Gaussia Luciferase Assay In Vitro

To conduct the luciferase assay of the cell culture supernatant, 20 μL of supernatant was mixed with 50 μL of 1% Gaussia luciferase assay substrate (Beyotime, RG021M) per well in a black 96-well plate. After incubation for 10 min at room temperature, the bioluminescence was read by using a SpectraMax® iD5 plate reader (Molecular Devices, Sunnyvale, CA, USA) or an IVIS spectrum imager (PerkinElmer, Waltham, MA, USA), the settings of which were open filter, 8 binning, F/1 aperture control, and auto-exposure time. For the measurement of the bioluminescence of blood samples, 50–100 μL of mouse blood was collected from the orbital vein, left at 37 °C for half an hour, and then centrifuged at 2000 rpm for 5 min to collect the upper serum. Later, 20 μL of serum was mixed with 50 μL of 1% Gaussia luciferase assay substrate per well in a black 96-well plate, and the bioluminescence was read using a microplate reader or an IVIS spectrum imager in the same way as in the process above.

### 4.14. Western Blotting

Protein samples of different groups were diluted to the same concentration using the BCA method (Beyotime, P0009) and separated by 4–20% gradient SDS-PAGE (Meilunbio, Dalian, China, MA0287) under the conditions of 80 V electrophoresis for 15 min and 120 V electrophoresis for 1 h. When transferring the protein from the gel to the nitrocellulose membrane (PALL, P-N66485) under the condition of 300 mA for 60 min was completed, the membrane was sealed with 5% skim milk (Solarbio, D8340) at room temperature for 1 h, and the primary antibody was added and incubated together at 4 °C on a shaker overnight. The membrane was washed three times with 0.1% TBST for 5 min each time the next day. After that, HRP-labeled secondary antibody (Zsbio, ZB2301/ZB2305) was added and incubated together at room temperature for 1 h, and the membrane was washed three times with 0.1% TBST for 5 min each time. Finally, blot bands were recorded using the A1600 system (GE Health, Chicago, IL, USA), and quantitative analysis was performed using ImageJ software. The antibodies and dilutions used were as follows: rabbit anti-BDNF antibody (Abcam, Cambridge, U.K., ab108319, 1:2000), mouse anti-Flag antibody (Sigma, F1804, 1:1000), goat anti-GFP antibody (Abcam, ab6673, 1:2000), rabbit anti-gaussia luciferase antibody (ThermoFisher, PA1-181, 1:1000) and rabbit anti-mouse IgG1 Fc antibody (SinoBiological, Beijing, China, 10690-T16, 1:1000).

### 4.15. RNA Sequencing

Total RNA was extracted using TRIzol reagent (Invitrogen, Carlsbad, CA, USA, 15596026CN) according to the manufacturer’s protocol. The RNA purity and quantity were evaluated using the NanoDrop 2000 spectrophotometer (Thermo Scientific, Waltham, MA, USA). The RNA integrity was assessed using the Agilent 2100 Bioanalyzer (Agilent Technologies, Santa Clara, CA, USA). Then, the libraries were constructed using the VAHTS Universal V6 RNA-seq Library Prep Kit according to the manufacturer’s instructions. The transcriptome sequencing and analysis were conducted by OE Biotech Co., Ltd. (Shanghai, China).

### 4.16. Statistical Analysis

All data were analyzed using Prism (GraphPad 8 software, LLC) or Excel (Microsoft). A Shapiro–Wilk test was first applied to determine whether the data were normally distributed. If the data were normally distributed, one-way or two-way ANOVA was used with Tukey’s multiple comparisons test in the comparison of three or more groups, or a nonparametric unpaired *t* test with a two-tailed *p* value was used in the comparison of two groups. Otherwise, a Mann–Whitney rank sum test was used in the comparison of two groups, or Kruskal–Wallis one-way ANOVA on ranks with post hoc Dunn’s test was used in the comparison of three or more groups. *p* < 0.05 indicates a statistically significant difference in the data and is represented by *; *p* < 0.01 indicates that the data have a very significant statistical difference and is represented by **; *p* < 0.001 means that the data have a very significant statistical difference, expressed by ***; and *p* < 0.0001 means that the data have a remarkably significant statistical difference and is represented by ****.

## Data Availability

All data are available in the main text.
